# Consolidation of Two Prominent Journals

**Published:** 1903-07

**Authors:** 


A Monthly Review of Medicine and Surgery.

EDITOR:

WILLIAM WARREN POTTER, M. D.

All communications, whether of a literary or business nature, books for review and exchanges should be addressed to the editor: 284 Franklin Street, Buffalo, N. Y.

Vol. XLII.—LVIII. JULY, 1903. No. 12.

### Consolidation of Two Prominent Journals.

DURING the past month the consolidation of the *New York Medical Journal* and the *Philadelphia Medical Journal* has been accomplished. The editor of the former, Dr. Frank P. Foster, who, indeed, becomes the editor of the united journals, announces the purposes of the union in an editorial article published in the issue of the amalgamated journals of June 20, 1903, in the following graceful manner:

In bringing about the consolidation the publishers have not been actuated solely by a desire to enlarge the subscription list, though they do not profess to have been unmindful of the advantage to be derived from such accretion. They have cherished the far higher purpose of combining and furnishing to an enlarged circle of readers all the features thought to be of special value in the two journals. We shall exert all possible efforts for the realisation of this purpose.

Before the consolidation the *New York Medical Journal* was free from all commercial influence, and so was the *Philadelphia Medical Journal*. Two journals more harmonious in their aims and methods do not exist, and it is most fitting, therefore, that they should so combine their resources as to further those aims and improve those methods to the utmost. This we believe to be wholly feasible under the unification of the two. Fortunately, the two cities, New York and Philadelphia, are of such ready access to each other that we apprehend no difficulty in dealing with medical matters of local interest in each city, especially as we shall maintain a Philadelphia office at which the editor will frequently be present. If New York is the larger of the two towns, and therefore presumably the scene of more events, we do not for a moment forget that Philadelphia is conspicuously glorious in the annals of medicine or that she is destined to be forever a leader in the progress of our profession.

We shall see to it that she is fittingly represented in our columns.

We have a few words in particular to say to the readers of the *Philadelphia Medical Journal*—a host of cultured and progressive physicians. They have done well to subscribe to such an excellent journal. Let us assure them that their favorite periodical is not to be merely absorbed—snuffed out, so to speak. Though it loses its distinctive title, it will perpetuate itself as an integral part of our united publication, even as a woman, when she marries, does indeed lose her father's name, but parts with not one whit of her individuality or of her influence. We have received from the accomplished editors of the *Philadelphia Medical Journal* a clean and able journal; we shall endeavor so to sustain its excellence as to merit the continuance of its patronage.

The time has arrived, in our view, when the aggregate number of medical journals should be reduced, and we have no doubt the action above delineated will prove advantageous to all parties concerned. In this spirit we beg to offer our congratulations to the editor and publisher of the consolidated journals referred to in the foregoing announcement.

